# Investigating psychotherapists’ attitudes towards artificial intelligence in psychotherapy

**DOI:** 10.1186/s40359-025-03071-7

**Published:** 2025-07-01

**Authors:** Julian Wagner, Anna-Sophia Schwind

**Affiliations:** 1 Department of Psychology, University of Trier, Trier, 54296 Germany; 2 Department of Applied Psychology, University of Applied Sciences Ravensburg-Weingarten, Weingarten, 88250 Germany

**Keywords:** Applications of artificial intelligence, Psychotherapist, Barriers, Implementation

## Abstract

**Background:**

The increasing prevalence of mental health disorders, compounded by a global shortage of psychotherapists, highlights the need for innovative solutions such as Artificial Intelligence (AI) or Machine Learning (ML) applications. These technologies have demonstrated potential in diagnostics, treatment personalization, and therapy optimization. However, their integration into psychotherapeutic practice requires understanding psychotherapists’ attitudes toward AI/ML, which remains underexplored. This study aims to investigate these attitudes, focusing on factors influencing AI acceptance and perceived usefulness.

**Methods:**

A cross-sectional survey was conducted among 181 licensed psychotherapists in Germany, recruited via the German Psychotherapeutical Association’s online directory. The survey assessed attitudes toward AI/ML, technical affinity, and perceptions of AI’s utility across psychotherapeutic tasks. Hierarchical regression analyses were used to identify predictors of AI acceptance.

**Results:**

Positive attitudes toward AI/ML were significantly predicted by its perceived usefulness in conducting diagnoses and creating personalized treatment plans. Empathic support, while rated lower in terms of enhancing therapy, was still a significant predictor across all groups. Technically affine therapists associated AI with benefits in diagnostics, whereas non-affine therapists emphasized empathic support and relapse prediction.

**Conclusion:**

Negative attitudes toward AI/ML application are discussed in a frame of fears of professional replacement and limited understanding of AI/ML technologies. Overall, 40% of the sample self-identified as not technically inclined, suggesting a knowledge gap in AI/ML that might influence attitudes. Education could emerge as a critical factor in addressing fears and uncertainties surrounding AI/ML. Emphasizing the irreplaceable human qualities of psychotherapists may also alleviate fears of obsolescence.

**Supplementary Information:**

The online version contains supplementary material available at 10.1186/s40359-025-03071-7.

## Theoretical background

Since the COVID-19 pandemic, the prevalence of mental health disorders has risen sharply, intensifying an already growing need for care [1]. This demand is further strained by a shortage of mental health professionals [2]. In response, technological advances have significantly shaped the evolution of mental health services [3]. A growing body of research has examined how Artificial Intelligence (AI) and Machine Learning (ML) can support psychotherapy processes [4]. AI refers to systems capable of autonomous learning and decision-making, mimicking human cognition [5], while ML specifically enables systems to improve performance by learning from data [6, 7]. These technologies are increasingly used to assist both psychotherapists and patients, with most research focusing on augmenting therapeutic processes [8].

Machine Learning (ML) is increasingly employed in psychiatry to support predictive analytics, particularly in forecasting treatment outcomes [9–11]. Within the framework of precision psychiatry, ML enables the tailoring of treatment based on individual characteristics such as genetics, environment, and life history [12, 13]. This is especially relevant given the limitations of standardized approaches in addressing the heterogeneity of mental disorders [14, 15]. A meta-analysis by Vieira et al. [16] showed that ML models predicted 74% of responses to Cognitive Behavioral Therapy (CBT), with variations depending on the diagnosis. Similarly, Delgadillo and Gonzalez Salas Duhne [17] demonstrated better treatment outcomes in depression when patients were matched to suitable treatments based on algorithmic recommendations. ML has also shown strong potential in predicting psychotherapy dropout and adherence, two factors known to impact clinical outcomes and healthcare costs [18]. Bennemann et al. [19] compared multiple algorithms and found that the best model predicted over 63% of CBT dropouts before the first session, outperforming traditional statistical models. Other studies extended this work to alcohol abuse treatment [20], and web-based interventions using language patterns from text data [21]. Gonzalez Salas Duhne et al. [22] further demonstrated that algorithm-based treatment matching can reduce dropout rates and enhance symptom improvement. Beyond psychotherapy, ML has been applied to the prediction and classification of various psychopathologies. For instance, Betz et al. [23] used childhood trauma data to predict depression, while Chavanne et al. [24] identified neuroimaging markers for anxiety. ML has also been used to detect psychosis [25] and predict the persistence or severity of mental disorders [26]. In the context of relapse prevention, deep learning methods utilizing neural networks have proven useful in monitoring conditions like schizophrenia, which are characterized by high recurrence rates [27–29]. Finally, ML has demonstrated significant utility in improving diagnostic procedures, particularly where existing classification systems face challenges due to symptom overlap and heterogeneity [30]. For example, Dolce et al. [31] used ML to streamline diagnostic questionnaires while maintaining accuracy. Other approaches integrate demographic, clinical, and behavioral data to enhance diagnostic precision across disorders such as depression [32, 33], anxiety [34], and attention deficit hyperactivity disorder (ADHD) [35].

While much research focuses on augmenting psychotherapists’ tasks, recent advances also aim to directly enhance therapeutic skills. Chatbots using natural language processing (NLP), a subset of ML focused on human language, can simulate client interactions. For example, Maurya [36] used ChatGPT to practice counseling, noting its empathic responses despite limitations in non-verbal cues. Since feedback is often costly and subjective [37], Tanana et al. developed a “clientbot” that simulates patients while providing expert-level feedback, supporting skill development. Beyond training, therapists continue refining interpersonal skills [38]. Tools using ML and NLP can analyze therapist language to assess behaviors [39, 40], and evaluate skills like empathy and active listening automatically [41]. Imel et al. also introduced a feedback system measuring motivational interviewing and session behaviors, with 90% of users indicating willingness to apply it in practice [38]. Automated coding further supports monitoring of therapeutic alliance and outcomes [42, 43], highlighting the potential of AI-based tools in improving therapy quality and effectiveness.

AI and ML applications not only support psychotherapists through predictive insights and skill development tools, chatbots are also increasingly integrated into patient-facing technologies. Since the creation of ELIZA, Weizenbaum’s [44] early natural language processing program simulating a Rogerian psychotherapist, conversational agents have evolved significantly. Modern systems like Woebot deliver CBT and have demonstrated effectiveness in reducing depression and anxiety symptoms in young adults [45]. A systematic review by Li et al. [46] identified AI-powered chatbots as useful across a range of therapeutic modalities, including CBT, ACT, and skills training. Lim et al. [47] further confirmed symptom improvements through chatbot-delivered psychotherapy, emphasizing the role of design in engagement. These findings suggest that conversational agents can positively impact mental and physical health, offering accessible education and targeted intervention.

Another promising patient-centered tool is Virtual Reality (VR), a computer-generated 3D environment simulating real-life experiences. A review by Emmelkamp and Meyerbröker [48] supports VR’s efficacy in treating conditions such as panic disorder, agoraphobia, PTSD, and autism. Personalization, essential to VR effectiveness, can be enhanced by integrating AI and ML, which allow for real-time assessment of user behavior, emotions, and cognition, supporting more tailored interventions [49].

AI is also increasingly embedded in digital therapeutics, defined as clinically validated software tools that assist patients in managing health conditions [50]. For example, Bain et al. [51] evaluated AiCure, an AI-driven platform using facial recognition to monitor medication adherence. Participants using AiCure showed a 25% improvement in adherence compared to controls, highlighting the broader potential of AI integration in healthcare delivery.

In conclusion, recent advances in AI have created new opportunities for enhancing mental health services by providing feedback and intervention support for clients, therapists, and supervisors [52]. These technologies may improve treatment quality and broaden access to care [4]. However, a significant gap remains between AI’s potential and its practical implementation [53]. Yusof et al.’s [54] HOT-fit framework identifies key barriers to adoption, including human acceptance, organizational readiness, and technological transparency. Aligning these elements with institutional routines is critical for successful integration [55]. The gap between AI-enhanced tools and their limited use in psychotherapy practice may stem from multiple barriers, with attitudes playing a critical role in technology adoption [56, 57]. While studies have explored AI/ML in psychology education [58] and how theoretical orientations influence acceptance [59], no research has directly examined psychotherapists’ views on the practical value of AI in daily clinical tasks.

Thus, this study investigated German psychotherapists’ attitudes toward AI acceptance as a potential barrier to the adoption of AI/ML in clinical practice. As noted by Allen [60], “Even the best technology will fail if the end users are not open to it” (p. 1). Accordingly, the research question is: What are psychotherapists’ attitudes toward AI/ML, and where do they perceive its strengths and weaknesses?

### Hypotheses

#### H1. Significantly more psychotherapists versus other non-psychotherapists believe that AI/ML cannot replace a psychotherapist

Doraiswamy et al. [61] found that psychiatrists generally do not expect AI/ML technologies to replace human clinicians in most critical functions, indicating that although some tasks may be supported by technology, the role of mental health professionals remains indispensable. Similarly, Sebri et al. [59] reported that psychotherapists generally hold negative attitudes toward AI conducting treatment. Given the current enthusiasm surrounding AI [62], it might appear to non-therapists that psychotherapy is a field suitable for AI integration.

#### H2. Research-based AI/ML applications explain psychotherapists’ attitudes towards AI acceptance significantly better than applications being discussed in research and demographic information.

Gado et al. [63] examined psychology students’ acceptance of AI and identified perceived usefulness and ease of use as the strongest predictors of their attitudes toward AI. Furthermore, other studies showed that perceived usefulness is a significant predictor of behavioral intention [64, 65]. To further investigate the relationship between perceived usefulness of AI applications and attitudes toward AI acceptance, the present study evaluates specific AI/ML applications with varying degrees of scientific validation: research-validated applications, applications in development, and general practice features. Research-based applications, such as those examined by Benfares et al. [32] and Bennemann et al. [19], have undergone rigorous study and demonstrated utility, thus receiving greater attention and should exerting a more substantial influence on attitudes toward AI acceptance than emerging applications still under consideration.

#### H3. Technically affine psychotherapists differ in their predictors explaining their attitude toward AI/ML applications compared to non-technical affine psychotherapists.

As proposed in hypothesis 2, research-based AI/ML applications are expected to be the strongest predictors of attitudes toward AI acceptance across the sample of psychotherapists. According to Karrer et al. [66], technical affinity, a personality trait characterized by positive attitudes, enthusiasm, and trust toward technology, is associated with higher interest, acceptance, and familiarity with technology, as well as greater experience and knowledge. Thus, technically affine therapists, being more knowledgeable about AI/ML technologies, are likely to have attitudes that correspond more closely with their evaluations of research-validated applications. Conversely, applications still in development are hypothesized to exert a lesser influence on AI acceptance attitudes, given their lower current relevance. For non-affine therapists, who tend to be less familiar with technological advancements, there is expected to be less differentiation between research-validated and less established applications. Consequently, attitudes toward AI acceptance in this group are shaped by a wider array of applications, potentially reducing the predictive influence of research-based applications alone.

## Methods

### Study sample

The initial study sample comprised 212 licensed psychotherapists; however, 31 cases with missing data were excluded, resulting in 181 valid cases. Power analyses were conducted for all hypotheses using G*Power (Version 3.1.9.7), indicating a required sample size of *N* = 194 to detect medium effect sizes across hypotheses. The sample included 53 males, 127 females, and one participant who did not report gender. The majority of psychotherapists were between 35 and 64 years old (82.4%), with the largest representation in the 55-64 age group (*n* = 68). The youngest age group (25-34) was the smallest (*n* = 5), followed by the oldest group (65+) with 27 participants. The average years of experience was 20.7 (*SD* = 11.8), ranging from one to 53 years. The mean annual patient load was 125 (*SD* = 198), with 50 patients being the most frequently reported value (13.8%). Technical affinity was distributed as follows: 59.7% identified as technically inclined, while 40.3% did not. Among psychotherapeutic approaches recognized by German health insurances, behavioral therapy was most prevalent (*n* = 143), followed by psychodynamic therapy (*n* = 29) and psychoanalytic therapy (*n* = 8); one participant reported practicing an unlisted approach. To ensure generalizability, after analysis of the sample’s descriptive characteristics, one participant who did not report their gender and one participant who did not report a psychotherapeutic orientation recognized by German public health insurances were excluded. The final sample included in the statistical analyses was *N* = 179.

### Procedure

Outpatient psychotherapists in Germany were identified through the German Psychotherapeutical Association (DPtV, Deutsche Psychotherapeuten Vereinigung) website to access a large, high-quality sample with available contact information. This platform was selected because it exclusively lists licensed, practicing psychotherapists, enhancing the reliability and validity of the study data. The website’s filtering options (e.g., state, location, treatment setting, health insurance) allowed for targeted sample selection. To ensure geographic diversity, large regions (with a radius of up to 200 km) encompassing various treatment settings and types of health insurance were selected, specifically including the federal states of Baden-Württemberg, Bavaria, Hamburg, Saxony-Anhalt, and North Rhine-Westphalia. Psychotherapists with publicly available email addresses listed on the DPtV website were included, resulting in 2,766 potential contacts. Email-based recruitment was selected for its efficiency in reaching a large number of professionals across geographically diverse regions. This approach is particularly well-suited for health research, where timely and cost-effective participant recruitment is critical [67]. Furthermore, leveraging a trusted professional association’s database increases the likelihood of recruiting qualified and contextually relevant participants. The survey, administered via the SoSci Survey web application, was sent to 2,607 addresses after 159 emails were found invalid or blocked. The survey link remained active for six weeks. Before participating in the survey, participants received detailed information about the purpose and objectives of the study, the voluntary nature of their participation, and the assurance of anonymity. They were also informed that they could withdraw from the study at any time without any consequences. Only after providing explicit consent to participate were they granted access to the questionnaire. The entire procedure adhered to the Declaration of Helsinki. In total, 212 psychotherapists participated with informed consent, with 181 completing the questionnaire.

### Survey instrument

Given the limited research on psychotherapists’ attitudes toward AI, a survey instrument was developed to examine their acceptance of AI and beliefs about the potential strengths and weaknesses of AI/ML. The instruments used by Blease et al. [68] and Sebri et al. [59] served as the primary framework for the survey and were translated to German. To ensure face validity, a licensed psychotherapist reviewed the survey instrument. The instrument comprised three sections: (1) sociodemographic information, (2) attitudes toward AI acceptance, and (3) ratings of the perceived usefulness of AI/ML applications across various psychotherapeutic tasks.

*Sociodemographic questions* covered gender and age recorded in five categories (24-34, 35-44, 45-55, 55-64, 65 and above; like Blease et al., [68]). The therapeutic approach was also captured, offering response options for all therapy modalities recognized by German statutory health insurance: behavioral, psychodynamic, psychoanalytic, systemic, and other [59]. Additionally, items assessing ‘years of experience’ and ‘number of patients per year’ [69] were included to enable analysis based on therapist experience. Finally, based on Rettinger et al. [70], participants were asked to self-assess their technical affinity on a dichotomous scale, selecting either ‘yes’ or ‘no’.

To evaluate *psychotherapists’ attitudes toward AI acceptance*, the questionnaire from Sebri et al. [59] was adapted. A brief introduction envisioning a future which role AI could perform in the therapeutical process (see Supplements) was presented at the beginning. Psychotherapists were then asked to rate specific characteristics of AI/ML technology. Sebri et al. [59] questionnaire is grounded in the Technology Acceptance Model (TAM) [71, 72], with “technology acceptance” replaced by “AI acceptance”. The AI characteristics that had to be rated included: utility (belief that the technology will improve treatment effectiveness and outcomes), ease of use (technology will be simple to use), personal comfort (usage will feel secure and effortless), positive expectations (AI will yield successful results), desirability, innovativeness, and feasibility of implementation. To capture distinct positive (4-6) or negative (1-3) attitudes, the present study employed a six-point Likert scale instead of the original format. Cronbach’s $\alpha$ was calculated, demonstrating high internal consistency with a reliability coefficient of .89, aligning with previous findings from technology acceptance questionnaires [65]. Item discrimination power ranged from *r* =.591 to.796, with most correlations exceeding .7 (*n* = 4), indicating that the items effectively differentiate between individuals with high and low levels of the characteristic under study.

The last three items in this part of the questionnaire addressed AI’s potential, adapted from the questionnaires by Blease et al. [68] and Doraiswamy et al. [61]. Psychotherapists were asked: (1) whether they believe the potential benefits of AI outweigh the risks, and (2) if they think AI could eventually replace a human psychotherapist. For both questions, response options were ‘yes’, ‘no’, and ‘uncertain’. For those agreeing with the second question, an additional question prompted them to estimate the timeframe for AI replacement, with response options of “0-4 years”, “5-10 years”, “11-25 years”, “25-50 years”, and “more than 50 years from now” [61].

**Fig. 1:**
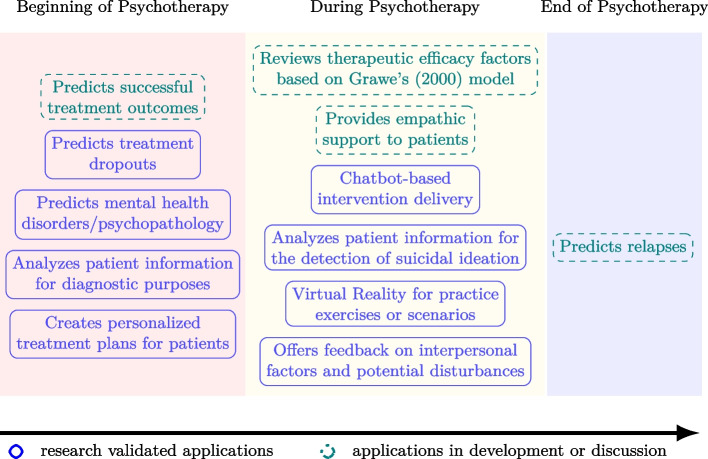
AI/ML applications across the psychotherapeutical process

The final section of the questionaire addressed the *perceived strengths and weaknesses of AI/ML integration* in core psychotherapeutic tasks, with items adapted from Blease et al. [68]. Participants were asked to evaluate whether these technologies enhance therapeutic tasks/applications or offer no added value. A six-point Likert scale, ranging from 1 (weakness) to 6 (strength), was used to rate the perceived usefulness of AI/ML integration. To facilitate intuitive responses and reduce cognitive load [73], only the scale endpoints were labeled, and an even-numbered scale was chosen to prompt directional choice. Items covered potential AI/ML applications across the therapeutic treatment process (see Fig. 1), all of which were either validated by previous research or discussed as promising developments.

### Data analysis

Statistical analyses were conducted using IBM SPSS Statistics (Version 29.0.1.0). To test Hypothesis 1, a ${\chi }^2$ Goodness-of-Fit Test was used to evaluate the frequency difference on the item concerning AI/ML replacing psychotherapists between psychotherapists and non-psychotherapists. Given an expected probability of AI replacement (*p* =.004; [74]) and thus a high probability of no replacement (*p* =.99), the third category - those expressing uncertainty - was excluded from the analysis (*n* = 9). As one cell (50%) had an expected frequency less than 5, an exact test was applied.

To test Hypothesis 2, a hierarchical multiple regression analysis was conducted to evaluate the predictive power of different variable sets on the criterion variable: the mean attitude score toward AI acceptance. The analysis proceeded in three stages. In the first block, general practice features were entered; the second block added AI applications currently in development or discussed in research, and the third block included research validated predictors (see Table 1). Each stage aimed to assess the incremental explanatory power of the newly added predictors after controlling for those in the preceding blocks.

**Table 1:** AI/ML application predictors entered in hierarchical regression analyses

Level 1: general practice features	Level 2: applications in development or discussion	Level 3: research validated applications
1. Number of treated patients per year	3. Predicts relapses	7. Analyzes patient information for diagnostic purposes
2. Years of professional experience	4. Predicts successful treatment outcomes	8. Analyzes patient information for the detection of suicidal ideation
	5. Reviews therapeutic efficacy factors based on Grawe’s (2000) model	9. Predicts mental health disorders/psychopathology
	6. Provides empathic support to patients	10. Predicts treatment dropouts
		11. Creates personalized treatment plans for patients
		12. Offers feedback on interpersonal factors and potential disturbances
		13. Chatbot-based intervention delivery
		14. Virtual Reality for practice exercises or scenarios

Model fit diagnostics utilized the Akaike Information Criterion (AIC), which measures the relative quality of the model based on a set of predictors, with lower AIC values indicating better fit. Multicollinearity was assessed via the Variance Inflation Factor (VIF), with VIF values below 10 deemed acceptable, indicating minimal multicollinearity among predictors and ensuring stable and interpretable regression coefficients. To test Hypothesis 3, separate regression analyses were conducted based on technology affinity levels (no/yes) after successfully evaluating the predictive impact of AI application possibilities on attitudes toward AI acceptance.

## Results

### Results of descriptive statistics analysis

Table 2 reveals that the majority of applications were evaluated with a mean rating below 3.5, reflecting a limited perceived utility within psychotherapeutic practice. The highest-rated applications demonstrated potential in employing Virtual Reality for exercising real-time scenarios, utilizing patient data for diagnostic purposes, and predicting mental health disorders. In contrast, applications integrating artificial intelligence and machine learning were rated as least effective in facilitating empathic support, providing feedback on interpersonal dynamics, and delivering interventions through chatbot interfaces.

**Table 2:** Mean, standard deviation and confidence interval statistics for AI/ML applications

AI/ML Applications	*M*	*SD*	*CI*
Predicts successful treatment outcomes	2.77	1.26	[2.58, 2.95]
Predicts treatment dropouts	2.88	1.34	[2.68, 3.07]
Predicts mental health disorders/psychopathology	3.13	1.40	[2.92, 3.33]
Analyzes patient information for diagnostic purposes	3.97	1.43	[3.79, 4.20]
Creates personalized treatment plans for patients	2.99	1.48	[2.77, 3.21]
Reviews therapeutic efficacy factors based on Grawe’s (2000) model	2.97	1.37	[2.76, 3.16]
Provides empathic support to patients	1.77	1.04	[1.61, 1.92]
Chatbot-based intervention delivery	2.67	1.27	[2.48, 2.85]
Analyzes patient information for the detection of suicidal ideation	2.96	1.31	[2.76, 3.15]
Virtual Reality for practice exercises or scenarios	4.19	1.48	[3.97, 4.40]
Offers feedback on interpersonal factors and potential disturbances	2.35	1.25	[2.16, 2.52]
Predicts relapses	2.93	1.30	[2.73, 3.11]

*N* = 179; *M* = mean; *SD* = standard deviation; *CI* = confidence interval (95% confidence interval)

Technical affine psychotherapists evaluated most aspects of AI acceptance below 3.5, as shown in Table 3. The only exception was the innovativity item, which received a higher rating. Among all aspects, the implementation and utility of AI were rated the highest, while usability and desirability were rated the lowest. Additionally, Table 3 highlights the attitudes of non-technical affine psychotherapists toward AI acceptance. None of the items received ratings above 3.5, indicating a generally negative attitude. Consistent with the technical affine psychotherapists, innovativity and implementation were rated the highest, whereas usage and desirability were rated the lowest.

**Table 3:** Mean, standard deviation and confidence interval statistics for aspects of the attitude towards AI acceptance of technically affine and non-affine psychotherapists

		Utility	Easiness of Use	Individuals’ Sensations	Desirable	Innovative	Realistic/Possible to Implement	Attitude Score
Technical Affine	*M*	2.81	2.34	2.80	2.73	3.90	3.08	2.94
	*SD*	1.52	1.30	1.42	1.53	1.55	1.52	1.21
	*CI*	[2.53, 3.10]	[2.09, 2.59]	[2.53, 3.07]	[2.44, 3.02]	[3.60, 4.19]	[2.79, 3.37]	[2.71, 3.18]
Technical Non-Affine	*M*	2.44	2.05	2.42	2.18	3.42	2.96	2.58
	*SD*	1.12	1.01	1.22	1.22	1.39	1.38	0.93
	*CI*	[2.18, 2.70]	[1.82, 2.29]	[2.14, 2.71]	[1.89, 2.46]	[3.10, 3.75]	[2.64, 3.28]	[2.36, 2.80]
Total	*M*	2.66	2.23	2.65	2.51	3.71	3.03	2.80
	*SD*	1.38	1.20	1.35	1.43	1.50	1.46	1.12
	*CI*	[2.46, 2.87]	[2.05, 2.40]	[2.45, 2.84]	[2.30, 2.72]	[3.49, 3.93]	[2.82, 3.25]	[2.63, 2.96]

Technical Affine *n* = 106; Technical Non-Affine *n* = 73; *N* = 179; *M* = mean; *SD* = standard deviation; *CI* = confidence interval (95% confidence interval)

### Results for H1

There was no significant difference between the expected and actual frequency of the item regarding AI replacing psychotherapists, ${\chi }^2$ (1, 172) =.092, *p* = 1.

### Results for H2 and H3

Before performing the multiple regression, a correlation matrix was calculated to assess multicollinearity among the predictors. All correlation values were below 0.8, giving a first impression that multicollinearity was not an issue for the analysis. Results showed that Model selection favored Model 3 (Table 4), which explained 54.4% of the variance in the mean attitude towards AI score (adjusted *R*^2^ = 0.544; *R*^2^ = 0.580). The overall model fit was confirmed through the ANOVA test for regression, which revealed a significant result: *F*(14, 164) = 16.167, *p* <.001, indicating that the model reliably predicted the mean AI attitude score. An additional indicator of Model 3’s superior fit was the consistent decrease in the Akaike Information Criterion (AIC) from Model 1 to Model 3, alongside continuous, significant changes in the *F*-value.

**Table 4:** Model summary for testing hypothesis 2

Model	*R*-Squared	Adjusted *R*-Squared	Standard Error of Estimate	Change in *R*-Squared	Change in *F*	Significance of Change in *F*	Akaike Information Criterion
1	.006	-.006	1.126	.006	0.499	>.05	45.452
2	.480	.462	0.823	.475	39.268	<.001	−62.679
3	.580	.544	0.758	.100	4.849	<.001	−84.754

*N* = 179; *R*-Squared = explained variance of the model; *F* = test statistic; Significance of Change presented as *p*-value

For model 3 several predictors contributed significantly to the model (Table 5). Thereby item ‘creates personalized treatment plans for patients’ had the strongest influence, with a standardized beta coefficient of .176 and a significant *t*-value of *t* = 3.202, *p* = .002, indicating a strong positive relationship with the mean AI attitude score. Similarly, items ‘provides empathic support to patient’ ($\beta$ = .206., *t* = 2.734, *p* = .007) and “analyzes patient information for diagnostic purposes” ($\beta$ = .118, *t* = 2.270, *p* = .025), also showed significant positive associations with the outcome variable but less strongly. The item “predicts mental health disorders/psychopathology” ($\beta$ = .129, *t* = 1.934, *p* = .055) narrowly missed the threshold for statistical significance. All other predictors also failed to reach significance, suggesting that these variables did not substantially contribute to explaining attitudes toward AI as beneficial in the therapeutic process. Further model fit analyzes revealed that the VIF values for all predictors were below 2.5, indicating an absence of multicollinearity concerns in the model. Outlier analysis showed that 95% of the cases had standardized residuals within ±2, and 99% of the cases fell within ±2.5. Five potential outliers were identified, but both had Cook’s distance values below 1, and their values were within twice the average, meaning these outliers did not necessitate exclusion from the model. Finally, residual analysis confirmed that the residuals were normally distributed, validating the assumptions of the regression analysis.

**Table 5:** Regression coefficients statistics for mean attitude toward AI/ML applications

Predictors	Beta	Standardized Beta	*t*-Value	Significance	Confidence Interval	
					Lower Bound	Higher Bound
Constant	.316		1.342	.181	-.149	.782
Number of treated patients per year	3.336E-6	.001	.001	.991	-.001	.001
Years of professional experience	.000	-.004	−0.078	.938	-.011	.010
Predicts relapses	.130	.151	1.666	.098	-.024	.284
Predicts successful treatment outcomes	.028	.031	.339	.735	-.135	.191
Reviews therapeutic efficacy factors based on Grawe’s (2000) model	-.032	-.038	-.546	.586	-.146	.083
Provides empathic support to patients	.206	.191	2.734	**.007**	.057	.355
Analyze patient information to conduct diagnosis	.118	.148	2.270	**.025**	.015	.220
Analyzes patient information for the detection of suicidal ideation	.025	.029	.420	.675	-.093	.143
Predicts mental health disorders/psychopathology	.129	.161	1.934	.055	-.003	.261
Predicts treatment dropouts	-.047	-.005	-.583	.561	-.204	.111
Creates personalized treatment plans for patients	.176	.231	3.202	**.002**	.067	.284
Offers feedback on interpersonal factors and potential disturbances	.066	.073	1.021	.309	-.062	.195
Chatbot-based intervention delivery	.068	.077	1.055	.293	-.059	.196
VR for practice exercises or scenarios	.020	.026	.384	.701	-.081	.568

*N* = 179; Significance presented as *p*-value, with values $\le \ .05$ highlighted; 95% confidence interval

For the subset of participants without technical affinity (*n* = 73), a regression analysis was conducted to determine whether the hierarchical regression results could be generalized or if motivational factors uniquely influenced the attitude towards AI applications. Examining non-technic affine psychotherapists it was found that model 2 accounted for 36% of the variance in attitude ($R^2$ =.363, adjusted $R^2$ =.305; Table 6), with an overall significant model fit of *F*(6, 66) = 6.268, *p* <.001. Interestingly, unlike in the initial regression analysis, model 3 contributed no predictive value. Among the predictors, the item ‘provides empathic support for patients’ stands out as a significant positive predictor ($\beta$ =.299, *t* = 2.733, *p* =.009). The item ‘predicts relapses’ is another predictor that showed significance ($\beta$ =.202, *t* = 2.013, *p* =.048). The remaining predictors show non-significant *p*-values, suggesting a limited direct impact on AI attitudes within this model. The VIF values for each predictor remain below 1.8, indicating an acceptable level of multicollinearity and affirming the independence of each predictor’s contribution to the regression model.

**Table 6:** Model summary for regression analysis of technically affine and non-affine psychotherapists

	Model	*R*-Squared	Adjusted *R*-Squared	Standard Error of Estimate	Change in *R*-Squared	Change in *F*	Significance of Change in *F*	Akaike Information Criterion
Technical Affine	1	.006	-.013	1.228	.006	0.328	>.05	46.535
	2	.559	.532	0.834	.552	30.967	<.001	−31.480
	3	.661	.609	0.763	.102	3.434	.002	−43.447
Technical Non-Affine	1	.043	.015	0.922	.043	1.567	>.05	−8.851
	2	.363	.305	0.775	.320	8.292	<.001	−30.575
	3	.470	.342	0.754	.107	1.463	>.05	−27.996

Technical Affine *n* = 106; Technical Non-Affine *n* = 73; *R*-Squared = explained variance of the model; *F* = test statistic; Significance of Change presented as *p*-value

In contrast, psychotherapists who indicated technical affinity (*n* = 106) demonstrated a stronger model explaining a notably higher 66% of the variance in AI attitude. Consistent with the initial regression analysis, Model 3 provided the best fit, *F*(14, 91) = 12.672, *p* <.001 (see Table 6). Notably, in the sample of technical affine psychotherapists item ‘analyses patient information for diagnosis purposes’ has the highest standardized beta ($\beta$ =.205, *t* = 2.233, *p* =.028), followed by item “provides empathic support to patients’ ($\beta$ =.191, *t* = 2.030, *p* =.045). All other predictors showed non-significant *p*-values, suggesting they may not directly impact attitudes within this context. The VIF values across all predictors remain under 3.6, indicating minimal multicollinearity concerns but affirming the robustness of the model.

## Discussion

The study aimed to explore licensed psychotherapists’ perceptions of AI/ML applications in therapeutic contexts. The findings offer a nuanced understanding of how research-based, AI/ML-specific, and practice-oriented variables influence attitudes toward diagnostic and treatment methodologies. Notably, predictors associated with personalized care and the diagnostic process emerged as the most significant, underscoring their critical role in shaping professional perspectives within psychological practice. Furthermore, the results highlight the relevance of the personality trait of technological affinity. Psychotherapists with a higher interest in technology demonstrated a more sophisticated understanding and greater acceptance of AI applications, as evidenced by the additional explained variance. This suggests that technological affinity enhances the predictive value of AI/ML-related factors and may inform the development of targeted strategies for integrating AI/ML into psychological interventions.

The present study found no significant differences between psychotherapists and engineers regarding their assessment of whether psychotherapists will be replaced by AI-driven stand-alone applications in the near future. Although it is widely acknowledged that many professions may soon be impacted by AI integration [75], there is a lack of empirically rigorous studies specifically addressing the likelihood of psychotherapists being supplanted by AI, apart from the projections offered by Frey and Osborne [74]. Nevertheless, public discourse has highlighted psychotherapy as a potentially vulnerable profession. For instance, the German Ethics Council [76] stated: “Psychotherapy is one of the few areas of medicine in which AI-based systems are already largely or even completely replacing doctors and other healthcare professionals, at least in certain contexts and groups” (translated from German, p. 210). This assertion has been challenged by the German Society for Psychology (DGPs) [77], which published a counterargument titled “AI-based systems as a substitute for psychotherapy? A definite no!” [77]. The DGPs critique emphasized that current digital health applications are often misinterpreted as AI-driven, whereas they primarily rely on deterministic decision trees based on if-then logic. These systems lack the sophistication to constitute autonomous psychotherapy, as the current generation of such applications is “not particularly artificially intelligent” [78] (p. 2). Consequently, while many occupational domains may be susceptible to AI adoption, the prevailing view among researchers, the DGPs, and practicing psychotherapists is that psychotherapy does not face an imminent risk of being replaced by AI-driven systems. However, the suggestion by prominent moral authorities that the profession of psychotherapy could potentially be replaced by technical applications may leave a bitter aftertaste for psychotherapists. Such statements, even if not substantiated by robust empirical evidence, could influence public perception and professional confidence, raising concerns about the long-term implications for the identity and societal valuation of the psychotherapeutic profession.

The results of our hierarchical regression analysis indicate that two research-based factors significantly predict a more positive attitude toward AI applications in the psychotherapeutic process: their use in conducting diagnoses and in the personalization of treatment plans. Another factor, the prediction of psychopathology, approached but did not reach statistical significance. Notably, one item frequently discussed in the literature - AI’s potential to provide empathic treatment to patients [36, 79, 80] - emerged as having the second strongest predictive power. Thus hypothesis 2 therefore can partially be accepted. Given the ongoing debates and concerns surrounding the reliability of mental disorder diagnoses [81, 82], it is understandable that a more favorable attitude toward AI applications in psychotherapy aligns with their potential to enhance diagnostic accuracy. In psychotherapy, particularly in (cognitive) behavioral therapy settings, the accuracy of a diagnosis is pivotal, as it forms the foundation for disorder-specific treatment planning. If it is assumed that AI algorithms, through extensive training, can achieve significantly higher diagnostic accuracy than human practitioners (e.g., psychotherapists; [83]), this presents a dual benefit: for practitioners, improved treatment efficiency through more tailored intervention strategies, and for patients, greater confidence in receiving an accurate diagnosis regardless of the specific practitioner consulted. This advantage is amplified when considering the second significant predictor identified in the study - personalized treatment planning. For decades, psychotherapy research has aimed to uncover the mechanisms underlying therapeutic effectiveness, though conclusive evidence remains elusive. A foundational model proposed by Grawe [84] identifies five general effectiveness factors in psychotherapy: the therapeutic relationship, motivational clarification, problem actualization, resource activation, and problem solving. The latter four factors, traditionally, psychotherapists must assess within the initial sessions using a range of methods, such as interviews, symptom questionnaires, and performance or personality tests, and then integrate this information into a cohesive treatment plan. This process is time-intensive and cognitively demanding. An AI-based tool capable of synthesizing data from diverse sources could alleviate this burden by streamlining the evaluation process. Psychotherapists would then be able to focus more holistically and efficiently on implementing treatment goals. Furthermore, treatment planning guided by AI would align more closely with evidence-based effectiveness factors, enhancing both the precision and scientific grounding of therapeutic interventions. Interestingly, the item ‘reviews therapeutic efficacy factors based on Grawe’s [84] model’ was not identified as a potential AI/ML application in the present study. A plausible explanation for this finding could be the age distribution within the study sample. Effectiveness research has gained significant traction in recent years, and older therapists may have limited familiarity with contemporary advancements in assessment methodologies. This finding underscores the potential of AI/ML applications to facilitate a more holistic evaluation of the therapeutic process, thereby enhancing treatment quality and optimizing the overall effectiveness of psychotherapeutic interventions.

A notable predictor that diverges from the generally negative outlook on AI in psychotherapy is the item “provides patients with empathic support”. Interestingly, this item received the lowest rating in terms of its potential to enhance therapeutic work, as reflected in the descriptive results. We hypothesize that psychotherapists with a positive attitude toward AI applications perceive this advantage primarily outside the context of therapy sessions, such as in crisis interventions, guidance for therapeutic exercises, or addressing patients’ immediate needs. This interpretation aligns with recent research [85] indicating that disorder-specific chatbots can effectively facilitate smaller, targeted interventions, particularly for exercise-based therapeutic tasks. However, the application of chatbots as “empathic” therapist substitutes during times when the therapist is unavailable remains underexplored in the literature. This concept presents both opportunities and challenges. On the positive side, such tools could provide continuity in care when human resources are limited, for example, when patients only have access to one therapy session per week. In these cases, patients could continue working on their issues between sessions, and therapists could later review chatbot interactions to monitor progress or adapt treatment plans. Conversely, significant challenges must be addressed, particularly in scenarios involving acute mental health crises, such as suicidal ideation or psychotic episodes. Questions of liability and safety become paramount in such cases, as chatbots lack the capacity to manage high-risk situations adequately [86, 87]. Furthermore, concerns surrounding data protection and patient confidentiality present additional ethical and practical barriers to implementing such technologies [88]. These complexities highlight the need for continued research and careful consideration of the role of AI-driven tools in providing empathic support within psychotherapeutic contexts.

Interestingly, none of the other recorded predictors demonstrated significant influence on attitudes toward AI applications. This discrepancy highlights a divergence between the advancements in research and their practical implementation. Attitudes and convictions regarding the necessity and usefulness of AI tools appear to be significantly more favorable within research contexts than in clinical practice. Several factors may contribute to this gap. Practical barriers, such as operational technical challenges and concerns about the error-prone nature of AI tools, play a role [89]. Additionally, a psychological defense mechanism among psychotherapists may underlie resistance to AI adoption. As noted earlier, statements like those from the German Ethics Council, which suggest that psychotherapy could be a profession vulnerable to replacement by AI applications, can elicit fear. This fear may activate functional or dysfunctional coping strategies aimed at regulating anxiety. A functional response within the context of this study would involve critically engaging with the potential benefits and limitations of AI tools to inform their appropriate use. However, if psychotherapists perceive AI-powered tools as a threat to their professional relevance, exacerbated by press coverage, it becomes more adaptive, in the short term, to reject the “enemy” outright. This defensive stance may manifest as a rejection of AI applications within the therapeutic process. Such resistance is further reinforced by pre-existing negative attitudes toward technology [89], reducing behavioral intentions to engage with or adopt AI tools. Davis’s Technology Acceptance Model (TAM) [64] provides a foundational framework for understanding behavioral intention to use technology, positing that attitudes toward technology are primarily influenced by its perceived usefulness and ease of use. Complementing this, Ajzen’s Theory of Planned Behavior [90] identifies three determinants of behavior: self-efficacy, subjective norms, and attitudes. Among these, attitudes have been shown to exert the strongest influence on behavior [91]. Therefore, negative attitudes toward AI/ML-enhanced applications are likely to hinder their adoption by psychotherapists, irrespective of the potential benefits these technologies might offer.

The results indicate that the perceived potential of AI applications is closely associated with technology affinity. The study provided greater insight into the attitudes of technology-affine psychotherapists compared to their non-technology-affine counterparts. Despite their differences, both groups consistently included the item “provides empathic support for patients” as a significant predictor of attitudes toward AI applications. However, their perspectives diverge in specific areas: therapists with a higher technology affinity perceived AI as particularly beneficial in the diagnostic process, whereas therapists with lower technology affinity emphasized its potential utility in predicting relapses. These findings support the confirmation of Hypothesis 3, underscoring the nuanced influence of technological orientation on psychotherapists’ attitudes toward AI-enhanced tools. Approximately 40% of the sample identified themselves as not technically inclined, suggesting that nearly half of the study participants lack substantial knowledge about AI/ML technologies. This aligns with findings that knowledge about AI/ML is rarely incorporated into psychotherapist training programs [58]. A lack of education in this area fosters uncertainty regarding the utility of AI/ML and may contribute to fears of professional replacement [92]. Such fears may also stem from doubts about one’s technical competencies [59]. To address these concerns, integrating AI/ML education into psychotherapy training programs is essential, as proposed by Blease et al. [58]. Their research demonstrated a significant positive correlation between increased hours of AI/ML education and favorable attitudes toward AI/ML integration. Educational interventions could be incorporated into university curricula, licensure processes, or ongoing professional development courses. These programs should focus on demonstrating how AI/ML can be applied to specific psychotherapeutic tasks and on teaching therapists how to effectively use these tools [93]. Moreover, it is crucial to emphasize the irreplaceable qualities of human psychotherapists, such as their capacity for deep understanding of mental illnesses and the accompanying emotional nuances [61]. Reinforcing the unique and indispensable role of psychotherapists can help alleviate fears of obsolescence and foster confidence in their professional value.

Finally, another potential source of bias in the results is the unbalanced gender distribution. It could be argued that the higher proportion of female participants may lead to an overrepresentation of non-technology affinity and related attitudes. However, our analyses indicate that among women in the sample, the distribution of non-technology affinity versus technology affinity was relatively balanced (47.2% vs. 52.8%), with a slight tendency toward greater technology affinity. In 2024, the overall gender distribution of licensed outpatient psychotherapists in Germany was 77.7% female and 22.3% male [94], which closely aligns with the composition of our sample (71% female and 29% male). Thus, the gender bias present in the sample reflects the actual demographics of outpatient psychotherapists in Germany, supporting the generalizability of our findings. Furthermore, the gender distribution among current psychology students, who represent future psychotherapists, also appears stable [95].

In conclusion, education is presumed to play a pivotal role in familiarizing psychotherapists with AI/ML, reducing uncertainty and fear, and facilitating the seamless integration of these technologies into psychotherapeutic practice. Furthermore, education empowers psychotherapists to actively contribute to the development of future AI/ML applications. As experts in delivering psychotherapy, psychotherapists possess valuable insights into the practical needs and utility of such technologies, guiding the creation of tools that are both relevant and effective. Their involvement in the developmental process not only ensures that AI/ML applications align with clinical realities but may also enhance compliance and acceptance in their future use [38]. This collaborative approach underscores the reciprocal benefits of integrating education and practitioner expertise in advancing AI/ML applications in psychotherapy.

### Limitations and future research

This study aimed to understand psychotherapists’ attitudes toward AI acceptance, with familiarity with the technology assessed solely through self-declared technical affinity. While this approach allowed for comparisons between technically affine and non-affine psychotherapists, which provided valuable insights into potential differences, it represents a limitation. Future studies should include a more precise assessment of AI/ML knowledge to better capture the nuances of familiarity with these technologies.

Another limitation lies in the study sample. To access a large sample with available contact details, the online directory of the German Psychotherapeutical Association was used to identify licensed practicing psychotherapists. However, the study predominantly reflects the perspectives of psychotherapists in outpatient (ambulant) practices, whose settings and processes differ from those in stationary (inpatient) psychotherapy settings. For example, outpatient therapists typically fund acquisitions like new software themselves and integrate these tools independently [96], whereas clinics often procure such resources for their employed therapists. This disparity suggests that the integration of costly new technologies may present distinct challenges across these settings. Therefore, future research should explore the opinions of therapists working in both ambulant and stationary settings to obtain a comprehensive understanding of attitudes toward AI/ML integration.

Additionally, future studies should investigate the perspectives of patients. Since the integration of AI/ML aims not only to support psychotherapists but also to enhance patient outcomes, understanding patient comfort with these technologies is essential. Expanding beyond quantitative analyses, qualitative research could also provide psychotherapists an opportunity to elaborate on their views, offering deeper insights into the factors influencing their attitudes toward AI/ML. Such mixed-method approaches would further enrich the understanding of the challenges and opportunities associated with integrating AI/ML into psychotherapeutic practice.

## Conclusion

This study provides valuable insights into psychotherapists’ attitudes toward AI/ML applications, emphasizing key predictors such as diagnostic utility and personalized treatment planning. Moreover, higher technical affinity appears to positively influence the perceived enhancement of AI tools in the therapeutic process. These findings highlight the critical role of education in reducing uncertainty, alleviating fears of professional obsolescence, and fostering AI acceptance. Integrating AI/ML education into psychotherapy training, licensing, and professional development programs can empower psychotherapists to confidently engage with and contribute to the development of AI tools. Future research should extend these findings by exploring attitudes in inpatient settings, assessing patient perspectives, and utilizing qualitative methodologies to provide a more comprehensive understanding of AI/ML integration. Addressing these areas will allow the field to harness AI’s potential to improve therapeutic outcomes while safeguarding the unique and irreplaceable human aspects of psychotherapeutic care.

## Supplementary Information

Supplementary Material 1.

## Data Availability

Data is provided within the manuscript. The datasets used and/or analysed during the current study are available from the corresponding author on reasonable request.

## Abbreviations

ADHD: Attention deficit hyperactivity disorder
AI: Artificial intelligence
AIC: Akaike information criterion
CI: Confidence interval
DGPs: German society for psychology
DPtV: German psychotherapeutical association
GLM: Generalized linear model
M: Mean
ML: Machine learning
NLP: Natural language processing
SD: Standard deviation
TAM: Technology acceptance model
VIF: Variance inflation factor
VR: Virtual reality

## References

[1] Winkler P, Formanek T, Mlada K, Kagstrom A, Mohrova Z, Mohr P, et al. Increase in prevalence of current mental disorders in the context of COVID-19: analysis of repeated nationwide cross-sectional surveys. Epidemiol Psychiatr Sci. 2020;29:e173. 10.1017/S2045796020000888. 32988427 10.1017/S2045796020000888 PMC7573458

[2] World Health Organization. Mental Health ATLAS 2017. World Health Organization; 2018. https://www.who.int/publications-detail-redirect/9789241514019. Accessed 15 June 2024.

[3] Hermes ED, Lyon AR, Schueller SM, Glass JE. Measuring the Implementation of Behavioral Intervention Technologies: Recharacterization of Established Outcomes. J Med Internet Res. 2019;21(1):e11752. 10.2196/11752. 30681966 10.2196/11752 PMC6367669

[4] Rebelo AD, Verboom DE, dos Santos NR, de Graaf JW. The impact of artificial intelligence on the tasks of mental healthcare workers: A scoping review. Comput Hum Behav Artif Hum. 2023;1(2):100008. 10.1016/j.chbah.2023.100008.

[5] Russell SJ, Norvig P. Artificial Intelligence: A Modern Approach, Global Edition. 4th ed. Pearson; 2021. 10.1109/MSP.2017.2765202.

[6] Jordan MI, Mitchell TM. Machine learning: Trends, perspectives, and prospects. Science. 2015;349(6245):255–60. 10.1126/science.aaa8415. 26185243 10.1126/science.aaa8415

[7] Kellmeyer P. Big Brain Data: On the Responsible Use of Brain Data from Clinical and Consumer-Directed Neurotechnological Devices. Neuroethics. 2021;14:83–98. 10.1007/s12152-018-9371-x.34745382

[8] Gual-Montolio P, Jaén I, Martínez-Borba V, Castilla D, Suso-Ribera C. Using Artificial Intelligence to Enhance Ongoing Psychological Interventions for Emotional Problems in Real- or Close to Real-Time: A Systematic Review. Int J Environ Res Public Health. 2022;19(13):7737. 10.3390/ijerph19137737. 35805395 10.3390/ijerph19137737 PMC9266240

[9] Aafjes-van Doorn K, Kamsteeg C, Bate J, Aafjes M. A scoping review of machine learning in psychotherapy research. Psychother Res. 2021;31(1):92–116. 10.1080/10503307.2020.1808729. 32862761 10.1080/10503307.2020.1808729

[10] Gómez Penedo JM, Rubel J, Meglio M, Bornhauser L, Krieger T, Babl A, et al. Using machine learning algorithms to predict the effects of change processes in psychotherapy: Toward process-level treatment personalization. Psychotherapy. 2023;60(4):536–47. 10.1037/pst0000507. 37796546 10.1037/pst0000507

[11] Jankowsky K, Krakau L, Schroeders U, Zwerenz R, Beutel ME. Predicting treatment response using machine learning: A registered report. Br J Clin Psychol. 2024;63(2):137–55. 10.1111/bjc.12452. 38111213 10.1111/bjc.12452

[12] Bzdok D, Meyer-Lindenberg A. Machine Learning for Precision Psychiatry: Opportunities and Challenges. Biol Psychiatry Cogn Neurosci Neuroimaging. 2018;3(3):223–30. 10.1016/j.bpsc.2017.11.007. 29486863 10.1016/j.bpsc.2017.11.007

[13] Winter NR, Hahn T. Big Data, KI und Maschinenlernen auf dem Weg zur Precision-Psychiatry - wie verändern sie den therapeutischen Alltag? Fortschr Neurol Psychiatr. 2020;88(12):786–93. 10.1055/a-1234-6247. 32998163 10.1055/a-1234-6247

[14] Dilling H, Harald JF. Taschenführer zur ICD-10-Klassifikation psychischer Störungen. 9th ed. Bern: Hogrefe AG; 2019.

[15] Sajjadian M, Lam RW, Milev R, Rotzinger S, Frey BN, Soares CN, et al. Machine learning in the prediction of depression treatment outcomes: a systematic review and meta-analysis. Psychol Med. 2021;51(16):2742–51. 10.1017/S0033291721003871. 35575607 10.1017/S0033291721003871

[16] Vieira S, Liang X, Guiomar R, Mechelli A. Can we predict who will benefit from cognitive-behavioural therapy? A systematic review and meta-analysis of machine learning studies. Clin Psychol Rev. 2022;97:102193. 10.1016/j.cpr.2022.102193. 35995023 10.1016/j.cpr.2022.102193

[17] Delgadillo J, Gonzalez Salas Duhne P. Targeted prescription of cognitive–behavioral therapy versus person-centered counseling for depression using a machine learning approach. J Consult Clin Psychol. 2020;88(1):14–24. 10.1037/ccp0000476. 31841021 10.1037/ccp0000476

[18] Giesemann J, Delgadillo J, Schwartz B, Bennemann B, Lutz W. Predicting dropout from psychological treatment using different machine learning algorithms, resampling methods, and sample sizes. Psychother Res. 2023;33(6):683–95. 10.1080/10503307.2022.2161432. 36669124 10.1080/10503307.2022.2161432

[19] Bennemann B, Schwartz B, Giesemann J, Lutz W. Predicting patients who will drop out of out-patient psychotherapy using machine learning algorithms. Br J Psychiatr. 2022;220(4):192–201. 10.1192/bjp.2022.17. 10.1192/bjp.2022.17 35177132

[20] Cavicchioli M, Calesella F, Cazzetta S, Mariagrazia M, Ogliari A, Maffei C, et al. Investigating predictive factors of dialectical behavior therapy skills training efficacy for alcohol and concurrent substance use disorders: A machine learning study. Drug Alcohol Depend. 2021;224:108723. 10.1016/j.drugalcdep.2021.108723. 33965687 10.1016/j.drugalcdep.2021.108723

[21] Smink WAC, Sools AM, Postel MG, Tjong Kim Sang E, Elfrink A, Libbertz-Mohr LB, et al. Analysis of the Emails From the Dutch Web-Based Intervention “Alcohol de Baas’’: Assessment of Early Indications of Drop-Out in an Online Alcohol Abuse Intervention. Front Psychiatry. 2021;12:575931. 10.3389/fpsyt.2021.575931. 34975551 10.3389/fpsyt.2021.575931 PMC8714780

[22] Gonzalez Salas Duhne P, Delgadillo J, Lutz W. Predicting early dropout in online versus face-to-face guided self-help: A machine learning approach. Behav Res Therapy. 2022;159:104200. 10.1016/j.brat.2022.104200. 10.1016/j.brat.2022.104200 36244300

[23] Betz LT, Rosen M, Salokangas RKR, Kambeitz J. Disentangling the impact of childhood abuse and neglect on depressive affect in adulthood: A machine learning approach in a general population sample. J Affect Disord. 2022;315(1):17–26. 10.1016/j.jad.2022.07.042. 35882299 10.1016/j.jad.2022.07.042

[24] Chavanne AV, Paillère Martinot ML, Penttilä J, et al. Anxiety onset in adolescents: a machine-learning prediction. Mol Psychiatry. 2023;28:639–46. 10.1038/s41380-022-01840-z. 36481929 10.1038/s41380-022-01840-z PMC9908534

[25] Koutsouleris N, Dwyer DB, Degenhardt F, Maj C, Urquijo-Castro MF, Sanfelici R, et al. Multimodal Machine Learning Workflows for Prediction of Psychosis in Patients With Clinical High-Risk Syndromes and Recent-Onset Depression. JAMA Psychiatry. 2021;78(2):195–209. 10.1001/jamapsychiatry.2020.3604. 33263726 10.1001/jamapsychiatry.2020.3604 PMC7711566

[26] Kessler RC, van Loo HM, Wardenaar KJ, Bossarte RM, Brenner LA, Cai T, et al. Testing a machine-learning algorithm to predict the persistence and severity of major depressive disorder from baseline self-reports. Mol Psychiatry. 2016;21:1366–71. 10.1038/mp.2015.198. 26728563 10.1038/mp.2015.198 PMC4935654

[27] LeCun Y, Bengio Y, Hinton G. Deep learning. Nature. 2015;521(7553):436–44. 10.1038/nature14539. 26017442 10.1038/nature14539

[28] Robinson D, Woerner MG, Alvir JMJ, Bilder R, Goldman R, Geisler S, et al. Predictors of Relapse Following Response From a First Episode of Schizophrenia or Schizoaffective Disorder. Arch Gen Psychiatr. 1999;56(3):241–7. 10.1001/archpsyc.56.3.241. 10078501 10.1001/archpsyc.56.3.241

[29] Barnes TRE, Leeson VC, Mutsatsa SH, Watt HC, Hutton SB, Joyce EM. Duration of untreated psychosis and social function: 1-year follow-up study of first-episode schizophrenia. Br J Psychiatry. 2008;193(3):203–9. 10.1192/bjp.bp.108.049718. 18757977 10.1192/bjp.bp.108.049718 PMC2576506

[30] Ghosh CC, McVicar D, Davidson G, Shannon C, Armour C. What can we learn about the psychiatric diagnostic categories by analysing patients’ lived experiences with Machine-Learning? BMC Psychiatry. 2022;22(1):427. 10.1186/s12888-022-03984-2. 35751077 10.1186/s12888-022-03984-2 PMC9233399

[31] Dolce P, Marocco D, Maldonato MN, Sperandeo R. Toward a Machine Learning Predictive-Oriented Approach to Complement Explanatory Modeling. An Application for Evaluating Psychopathological Traits Based on Affective Neurosciences and Phenomenology. Front Psychol. 2020;11:446. 10.3389/fpsyg.2020.00446. 32265781 10.3389/fpsyg.2020.00446 PMC7105860

[32] Benfares C, Akhrif O, El Idrissi YEB, Hamid K. A clinical support system for classification and prediction of depression using machine learning methods. Comput Intell. 2021;37(4):1619–32. 10.1111/coin.12377.

[33] Choi B, Shim G, Jeong B, Jo S. Data-driven analysis using multiple self-report questionnaires to identify college students at high risk of depressive disorder. Sci Rep. 2020;10:7867. 10.1038/s41598-020-64709-7. 32398788 10.1038/s41598-020-64709-7 PMC7217968

[34] Bhatnagar S, Agarwal J, Sharma OR. Detection and classification of anxiety in university students through the application of machine learning. Procedia Comput Sci. 2023;218:1542–50. 10.1016/j.procs.2023.01.132.

[35] Mikolas P, Vahid A, Bernardoni F, Süß M, Martini J, Beste C, et al. Training a machine learning classifier to identify ADHD based on real-world clinical data from medical records - Scientific Reports. Sci Rep. 2022;12:12934. 10.1038/s41598-022-17126-x. 35902654 10.1038/s41598-022-17126-x PMC9334289

[36] Maurya RK. A qualitative content analysis of ChatGPT’s client simulation role-play for practising counselling skills. Couns Psychother Res. 2024;24(2):614–30. 10.1002/capr.12699.

[37] Tanana MJ, Soma CS, Srikumar V, Atkins DC, Imel ZE. Development and Evaluation of ClientBot: Patient-Like Conversational Agent to Train Basic Counseling Skills. J Med Internet Res. 2019;21(7):e12529. 10.2196/12529. 31309929 10.2196/12529 PMC6662153

[38] Imel ZE, Pace BT, Soma CS, Tanana M, Hirsch T, Gibson J, et al. Design feasibility of an automated, machine-learning based feedback system for motivational interviewing. Psychotherapy. 2019;56(2):318–28. 10.1037/pst0000221. 30958018 10.1037/pst0000221 PMC11270535

[39] Xiao B, Imel ZE, Georgiou PG, Atkins DC, Narayanan SS. “Rate My Therapist’’: Automated Detection of Empathy in Drug and Alcohol Counseling via Speech and Language Processing. PLoS ONE. 2015;10(12):e0143055. 10.1371/journal.pone.0143055. 26630392 10.1371/journal.pone.0143055 PMC4668058

[40] Goldberg SB, Tanana M, Imel ZE, Atkins DC, Hill CE, Anderson T. Can a computer detect interpersonal skills? Using machine learning to scale up the Facilitative Interpersonal Skills task. Psychother Res. 2021;31(3):281–8. 10.1080/10503307.2020.1741047. 32172682 10.1080/10503307.2020.1741047 PMC7492408

[41] Zhang X, Tanana M, Weitzman L, Narayanan S, Atkins D, Imel Z. You never know what you are going to get: Large-scale assessment of therapists’ supportive counseling skill use. Psychotherapy. 2023;60(2):149–58. 10.1037/pst0000460. 36301302 10.1037/pst0000460 PMC10133410

[42] Atzil-Slonim D, Juravski D, Bar-Kalifa E, Gilboa-Schechtman E, Tuval-Mashiach R, Shapira N, et al. Using topic models to identify clients’ functioning levels and alliance ruptures in psychotherapy. Psychotherapy. 2021;58(2):324–39. 10.1037/pst0000362. 33734743 10.1037/pst0000362

[43] Ewbank MP, Cummins R, Tablan V, Catarino A, Buchholz S, Blackwell AD. Understanding the relationship between patient language and outcomes in internet-enabled cognitive behavioural therapy: A deep learning approach to automatic coding of session transcripts. Psychother Res. 2021;31(3):300–12. 10.1080/10503307.2020.1788740. 10.1080/10503307.2020.1788740 32619163

[44] Weizenbaum J. ELIZA - A Computer Program For the Study of Natural Language Communication Between Man And Machine. Commun ACM. 1966;9(1):36–45. 10.1145/365153.365168.

[45] Fitzpatrick K, Darcy A, Vierhile M. Delivering Cognitive Behavior Therapy to Young Adults With Symptoms of Depression and Anxiety Using a Fully Automated Conversational Agent (Woebot): A Randomized Controlled Trial. JMIR Ment Health. 2017;4(2):e19. 10.2196/mental.7785. 28588005 10.2196/mental.7785 PMC5478797

[46] Li Y, Liang S, Zhu B, Liu X, Li J, Chen D, et al. Feasibility and effectiveness of artificial intelligence-driven conversational agents in healthcare interventions: A systematic review of randomized controlled trials. Int J Nurs Stud. 2023;143:104494. 10.1016/j.ijnurstu.2023.104494. 37146391 10.1016/j.ijnurstu.2023.104494

[47] Lim SM, Shiau CWC, Cheng LJ, Lau Y. Chatbot-Delivered Psychotherapy for Adults With Depressive and Anxiety Symptoms: A Systematic Review and Meta-Regression. Behav Ther. 2022;53(2):334–47. 10.1016/j.beth.2021.09.007. 35227408 10.1016/j.beth.2021.09.007

[48] Emmelkamp PMG, Meyerbröker K. Virtual Reality Therapy in Mental Health. Ann Rev Clin Psychol. 2021;17:495–519. 10.1146/annurev-clinpsy-081219-115923. 33606946 10.1146/annurev-clinpsy-081219-115923

[49] Riva G, Wiederhold BK, Di Lernia D, Chirico A, Riva EFM, Mantovani F, et al. Virtual Reality meets Artificial Intelligence: The Emergence of Advanced Digital Therapeutics and Digital Biomarkers. Annual Review of CyberTherapy and Telemedicine. 2019;17:3–7. https://psycnet.apa.org/record/2020-34322-001.

[50] Taub CJ, Zion SR, Ream M, Ramiller A, Heathcote LC, Eich G, et al. Cognitive behavioral digital therapeutic effects on distress and quality of life in patients with cancer: National randomized controlled trial. J Consult Clin Psychol. 2024;92(11):727–41. 10.1037/ccp0000911. 39621368 10.1037/ccp0000911

[51] Bain EE, Shafner L, Walling DP, Othman AA, Chuang-Stein C, Hinkle J, et al. Use of a Novel Artificial Intelligence Platform on Mobile Devices to Assess Dosing Compliance in a Phase 2 Clinical Trial in Subjects With Schizophrenia. JMIR mHealth uHealth. 2017;5(2):e18. 10.2196/mhealth.7030. 28223265 10.2196/mhealth.7030 PMC5340925

[52] Stade B, Stirman SW, Ungar LH, Boland CL, Schwartz HA, Yaden DB, et al. Large language models could change the future of behavioral healthcare: A proposal for responsible development and evaluation. npj Ment Health Res. 2024;3:12. 10.1038/s44184-024-00056-z. 38609507 10.1038/s44184-024-00056-z PMC10987499

[53] Sendak MP, D’Arcy J, Kashyap S, Gao M, Nichols M, Corey K, et al. A Path for Translation of Machine Learning Products into Healthcare Delivery. EMJ. 2020. 10.33590/emjinnov/19-00172.

[54] Yusof MM, Papazafeiropoulou A, Paul RJ, Stergioulas LK. Investigating evaluation frameworks for health information systems. Int J Med Inform. 2008;77(6):377–85. 10.1016/j.ijmedinf.2007.08.004. 17904898 10.1016/j.ijmedinf.2007.08.004

[55] Willcocks LP. Evaluating the outcomes of Information systems plans-managing information technology evaluation: Techniques and Processes. In: Strategic Information Management: Challenges and Strategies in Managing Information Systems. Routledge; 1994. pp. 365–81.

[56] Brender J, Ammenwerth E, Nykänen P, Talmon J. Factors influencing success and failure of health informatics systems-a pilot Delphi study. Methods Inf Med. 2006;45(1):125–36. 10.1055/s-0038-1634049. 16482383

[57] Dingel J, Kleine AK, Cecil J, Sigl AL, Lermer E, Gaube S. Predictors of Health Care Practitioners’ Intention to Use AI-Enabled Clinical Decision Support Systems: Meta-Analysis Based on the Unified Theory of Acceptance and Use of Technology. J Med Internet Res. 2024;26(1):e57224. 10.2196/57224. 39102675 10.2196/57224 PMC11333871

[58] Blease C, Kharko A, Annoni M, Gaab J, Locher C. Machine Learning in Clinical Psychology and Psychotherapy Education: A Mixed Methods Pilot Survey of Postgraduate Students at a Swiss University. Front Public Health. 2021;9:623088. 10.3389/fpubh.2021.623088. 33898374 10.3389/fpubh.2021.623088 PMC8064116

[59] Sebri V, Pizzoli SFM, Savioni L, Triberti S. Artificial Intelligence in mental health: professionals’ attitudes towards AI as a psychotherapist. Ann Rev CyberTherapy Telemed. 2020;18(1):229–33.

[60] Allen S. Artificial Intelligence and the Future of Psychiatry. IEEE Pulse. 2020;11(3):2–6. 10.1109/MPULS.2020.2993657. 32559160 10.1109/MPULS.2020.2993657

[61] Doraiswamy PM, Blease C, Bodner K. Artificial intelligence and the future of psychiatry: Insights from a global physician survey. Artif Intell Med. 2020;102:101753. 10.1016/j.artmed.2019.101753. 31980092 10.1016/j.artmed.2019.101753

[62] Strange M. Three different types of AI hype in healthcare. AI Ethics. 2024;4:833–40. 10.1007/s43681-024-00465-y.

[63] Gado S, Kempen R, Lingelbach K, Bipp T. Artificial intelligence in psychology: How can we enable psychology students to accept and use artificial intelligence? Psychol Learn Teach. 2022;21(1):37–56. 10.1177/14757257211037149.

[64] Davis F. Perceived Usefulness, Perceived Ease of Use, and User Acceptance of Information Technology. MIS Q. 1989;13(3):319–39. 10.2307/249008.

[65] King W, He J. A meta-analysis of the Technology Acceptance Model. Inf Manag. 2006;43(6):740–55. 10.1016/j.im.2006.05.003.

[66] Karrer K, Glaser C, Clemens C, Bruder C. Technikaffinität erfassen – der Fragebogen TA-EG. In: Der Mensch im Mittelpunkt technischer Systeme. ZMMS Spektrum; 2009. pp. 196–201. 10.23668/psycharchives.12181.

[67] Ng MY, Olgin JE, Marcus GM, Lyles CR, Pletcher MJ. Email-Based Recruitment Into the Health eHeart Study: Cohort Analysis of Invited Eligible Patients. J Med Internet Res. 2023;25:e51238. 10.2196/51238. 38133910 10.2196/51238 PMC10770794

[68] Blease C, Bernstein MH, Gaab J, Kaptchuk TJ, Kossowsky J, Mandl KD, et al. Computerization and the future of primary care: A survey of general practitioners in the UK. PLoS ONE. 2018;13(12):e0207418. 10.1371/journal.pone.0207418. 30540791 10.1371/journal.pone.0207418 PMC6291067

[69] Békés V, Aafjes-van Doorn K, Zilcha-Mano S, Prout T, Hoffman L. Psychotherapists’ acceptance of telepsychotherapy during the COVID-19 pandemic: A machine learning approach. Clin Psychol Psychother. 2021;28(6):1403–15. 10.1002/cpp.2682. 34723404 10.1002/cpp.2682 PMC8652775

[70] Rettinger L, Klupper C, Werner F, Putz P. Changing attitudes towards teletherapy in Austrian therapists during the COVID-19 pandemic. J Telemed Telecare. 2023;29(5):406–14. 10.1177/1357633X20986038. 33430678 10.1177/1357633X20986038 PMC10195684

[71] Davis F. A Technology Acceptance Model for Empirically Testing New End-User Information Systems. Massachusetts Institute of Technology; 1986. http://hdl.handle.net/1721.1/15192.

[72] Tamilmani K, Rana N, Dwivedi Y. Consumer Acceptance and Use of Information Technology: A Meta-Analytic Evaluation of UTAUT2. Inf Syst Front. 2021;23:987–1005. 10.1007/s10796-020-10007-6.

[73] Krosnick JA, Fabrigar LR. Designing Rating Scales for Effective Measurement in Surveys. In: Survey Measurement and Process Quality. Wiley; 1997. pp. 141–64. 10.1002/9781118490013.ch6.

[74] Frey CB, Osborne MA. The future of employment: How susceptible are jobs to computerisation? Technol Forecast Soc Chang. 2017;114:254–80. 10.1016/j.techfore.2016.08.019.

[75] Nnamdi N, Ogunlade B, Abegunde B. An Evaluation of the Impact of Artificial Intelligence on Socio-Economic Human Rights: A Discourse on Automation and Job Loss. Scholars Int J Law Crime Justice. 2023;6(10):508–21. 10.36348/sijlcj.2023.v06i10.001.

[76] Deutscher Ethikrat. Mensch und Maschine – Herausforderungen durch Künstliche Intelligenz. Deutscher Ethikrat; 2023. https://www.ethikrat.org/publikationen/stellungnahmen/mensch-und-maschine/. Accessed 12 Dec 2024.

[77] Deutsche Gesellschaft für Psychologie. Stellungnahme der Deutschen Gesellschaft für Psychologie auf die Schrift des Deutschen Ethikrates zu “Mensch und Maschine – Herausforderungen durch Künstliche Intelligenz”. Deutsche Gesellschaft für Psychologie; 2023. https://www.dgps.de/schwerpunkte/stellungnahmen-und-empfehlungen/stellungnahmen/details/ki-basiertesysteme-als-ersatz-fuer-psychotherapie-ein-eindeutiges-nein/. Accessed 12 Dec 2024.

[78] Ebert DD, Baumeister H. Digitale Gesundheitsinterventionen: Anwendungen in Therapie und Prävention. 1st ed. Berlin: Springer; 2023. 10.1007/978-3-662-65816-1.

[79] Brown JEH, Halpern J. AI chatbots cannot replace human interactions in the pursuit of more inclusive mental healthcare. SSM Ment Health. 2021;1:100017. 10.1016/j.ssmmh.2021.100017.

[80] Cheng SW, Chang CW, Chang WJ, Wang HW, Liang CS, Kishimoto T, et al. The now and future of ChatGPT and GPT in psychiatry. Psychiatry Clin Neurosci. 2023;77(11):592–6. 10.1111/pcn.13588. 37612880 10.1111/pcn.13588 PMC10952959

[81] Matarazzo JD. Behavioral immunogens and pathogens: Psychology’s newest challenge. Prof Psychol Res Pract. 1983;14(3):414–6. 10.1037/0735-7028.14.3.414.

[82] Reed GM, Sharan P, Rebello TJ, Keeley JW, Elena Medina-Mora M, Gureje O, et al. The ICD-11 developmental field study of reliability of diagnoses of high-burden mental disorders: results among adult patients in mental health settings of 13 countries. World Psychiatry. 2018;17(2):174–86. 10.1002/wps.20524. 29856568 10.1002/wps.20524 PMC5980511

[83] Schulte-Rüther M, Kulvicius T, Stroth S, Wolff N, Roessner V, Marschik PB, et al. Using machine learning to improve diagnostic assessment of ASD in the light of specific differential and co-occurring diagnoses. J Child Psychol Psychiatry. 2023;64(1):16–26. 10.1111/jcpp.13650. 35775235 10.1111/jcpp.13650

[84] Grawe K. Psychologische Therapie. 2nd ed. Hogrefe; 2000.

[85] Aggarwal A, Tam CC, Wu D, Li X, Qiao S. Artificial Intelligence-Based Chatbots for Promoting Health Behavioral Changes: Systematic Review. J Med Internet Res. 2023;25:e40789. 10.2196/40789. 36826990 10.2196/40789 PMC10007007

[86] De Freitas J, Uğuralp AK, Oğuz-Uğuralp Z, Puntoni S. Chatbots and mental health: Insights into the safety of generative AI. J Consum Psychol. 2024;34(3):481–91. 10.1002/jcpy.1393.

[87] Elyoseph Z, Levkovich I. Beyond human expertise: the promise and limitations of ChatGPT in suicide risk assessment. Front Psychiatry. 2023;14:1213141. 10.3389/fpsyt.2023.1213141. 37593450 10.3389/fpsyt.2023.1213141 PMC10427505

[88] Saeidnia HR, Fotami S, Lund B, Ghiasi N. Ethical Considerations in Artificial Intelligence Interventions for Mental Health and Well-Being: Ensuring Responsible Implementation and Impact. Soc Sci. 2024;13(7):1–15. 10.3390/socsci13070381.

[89] Kleine AK, Kokje E, Lermer E, Gaube S. Attitudes Toward the Adoption of 2 Artificial Intelligence-Enabled Mental Health Tools Among Prospective Psychotherapists: Cross-sectional Study. JMIR Hum Factors. 2023;10:e46859. 10.2196/46859. 37436801 10.2196/46859 PMC10372564

[90] Ajzen I. The theory of planned behavior. Organ Behav Hum Decis Process. 1991;50(2):179–211. 10.1016/0749-5978(91)90020-T.

[91] Conner M, Sparks P. The Theory of Planned Behaviour. In: Predicting Health Behaviour: Research and Practice with Social Cognition Models. vol. 2. Buckingham: Open University Press; 2005. pp. 121–62.

[92] Shen Y, Zhang X. The impact of artificial intelligence on employment: the role of virtual agglomeration. Humanit Soc Sci Commun. 2024;11:122. 10.1057/s41599-024-02647-9.

[93] Lutz W, Rubel JA, Schwartz B, Schilling V, Deisenhofer AK. Towards integrating personalized feedback research into clinical practice: Development of the Trier Treatment Navigator (TTN). Behav Res Ther. 2019;120:103438. 10.1016/j.brat.2019.103438. 31301550 10.1016/j.brat.2019.103438

[94] Kassenärztliche Bundesvereinigung. Statistische Informationen aus dem Bundesarztregister. Kassenärztliche Bundesvereinigung; 2024. https://www.kbv.de/media/sp/2023-12-31_BAR_Statistik.pdf. Accessed 14 May 2025.

[95] Antoni CH. Berufsfelder von Psychologinnen und Psychologen. Psychol Rundsch. 2024;75(2):119–33. 10.1026/0033-3042/a000668.

[96] Barker RL. The Business of Psychotherapy: Private Practice Administration for Therapists, Counselors, and Social Workers. Columbia University Press; 1982. https://archive.org/details/businessofpsycho0000bark/page/n5/mode/2up.

