# Supplementary material for: Investigating psychotherapists’ attitudes towards artificial intelligence in psychotherapy
Source: BMC Psychol. 2025 Jul 1;13:719. doi: 10.1186/s40359-025-03071-7 (PMC12220637; doi:10.1186/s40359-025-03071-7)
Supplement: Supplementary file 1 — Supplementary Material 1. [file 40359_2025_3071_MOESM1_ESM.docx]

**Survey instrument**

Welcome!

**Thank you for participating in this survey**.

This survey is part of my bachelor's thesis, which focuses on artificial intelligence and machine learning in the psychotherapeutic process.

Answering the questions will take approximately **10 minutes**.

Participation is **voluntary**, and all responses are stored **anonymously**. This ensures that no conclusions can be drawn about your identity. You can determine the survey at any given time without providing a reason and/or facing any disadvantages or negative consequences. "The collected data will be used exclusively for my bachelor's thesis and will be deleted from the survey provider's servers upon completion of data collection."

I have read the information above and agree to participate.

**I Socio-demographics**

**A. Gender**

male, female, diverse, no indication

**B. Age**

25-34; 35-44; 45-54; 55-64; 65 and over

**C. Theoretical orientation**

cognitive behavioral; psychodynamic; systemic-relational; other

**D. Years of experience**

**E. Number of patients per year**

**F. Technical affinity**

yes ; no

**II Attitudes**

Section 1:

*AI is able to use much data of patients and it is possible that, in the future, AI could replace a human psychotherapist by identifying the best interventions and monitoring patients on a daily basis. For example: AI monitors patients collecting and elaborating individual data related to their lifestyle and everyday symptoms; moreover, AI answers to patients’ questions and builds a psychotherapy intervention.*

Please rate the following traits of artificial intelligence from *lowest (1) to highest (6).*

*Options: 1-2-3-4-5-6*

- utility (belief that the technology will improve treatment effectiveness and outcomes), ease of use (technology will be simple to use)

- personal comfort (usage will feel secure and effortless)

- positive expectations (AI will yield successful results)

- desirability

- innovativeness

- feasibility of implementation

Section 2

*Options: “yes”, “no”, “uncertain”*

- Do potential benefits of AI/ML outweigh possible risks?

- Do you think AI-embedded technology can replace a human psychotherapist?

🡪 if yes, when? [*Options: 0-4 years; 5 to 10 years from now; 11 to 25 years from now; 26-50 years; more than 50 years from now*]

**III Strengths and Weaknesses**

You are given many tasks/applications relevant to psychotherapy. Please rate, whether the integration of artificial intelligence/machine learning could enhance (strength) or worsen (weakness) those tasks/applications.

Weakness: 1 = big deficit; 3 = slight deficit

Strength: 4 = slight advantage; 6 = big advantage

*Options: 1-2-3-4-5-6*

Applications for psychotherapists:

**Analyzation**

**-** analyzes patient information for diagnostic purposes

**-** analyzes patient information for the detection of suicidal ideation

**Generation**

**-** creates personalized treatment plans for patients

**Prediction**

- predicts successful treatment outcomes

**-** predicts treatment dropouts

- predicts mental health disorders/psychopathology

**-** predicts relapses

**Improvement of skills**

- reviews therapeutic efficacy factors based on Grawe’s (2000) model

- offers feedback on interpersonal factors and potential disturbances

Applications for patients:

**Chatbots and Apps**

- provides empathic support to patients

- Chatbot-based intervention delivery

- Virtual Reality for practice exercises or scenarios

**Thank you for your participation!**

Your responses have been saved, and you can now close the window.

If you have any questions, comments, or would like to receive the results, feel free to email me at: julian.wagner@hs-weingarten.de
